# Neural discriminability in rat lateral extrastriate cortex and deep but not superficial primary visual cortex correlates with shape discriminability

**DOI:** 10.3389/fncir.2015.00024

**Published:** 2015-05-20

**Authors:** Ben Vermaercke, Gert Van den Bergh, Florian Gerich, Hans Op de Beeck

**Affiliations:** ^1^Laboratory of Biological Psychology, Psychology and Educational Sciences, KU LeuvenLeuven, Belgium; ^2^Department for Molecular and Cellular Biology, Center for Brain Science, Harvard UniversityCambridge, MA, USA

**Keywords:** shape discrimination, rodent behavior, visual water maze, electrophysiological recording, population coding

## Abstract

Recent studies have revealed a surprising degree of functional specialization in rodent visual cortex. It is unknown to what degree this functional organization is related to the well-known hierarchical organization of the visual system in primates. We designed a study in rats that targets one of the hallmarks of the hierarchical object vision pathway in primates: selectivity for behaviorally relevant dimensions. We compared behavioral performance in a visual water maze with neural discriminability in five visual cortical areas. We tested behavioral discrimination in two independent batches of six rats using six pairs of shapes used previously to probe shape selectivity in monkey cortex (Lehky and Sereno, 2007). The relative difficulty (error rate) of shape pairs was strongly correlated between the two batches, indicating that some shape pairs were more difficult to discriminate than others. Then, we recorded in naive rats from five visual areas from primary visual cortex (V1) over areas LM, LI, LL, up to lateral occipito-temporal cortex (TO). Shape selectivity in the upper layers of V1, where the information enters cortex, correlated mostly with physical stimulus dissimilarity and not with behavioral performance. In contrast, neural discriminability in lower layers of all areas was strongly correlated with behavioral performance. These findings, in combination with the results from Vermaercke et al. (2014b), suggest that the functional specialization in rodent lateral visual cortex reflects a processing hierarchy resulting in the emergence of complex selectivity that is related to behaviorally relevant stimulus differences.

## Introduction

Interest in the use of rodents for research into the neurobiological underpinnings of vision has grown in recent years. While most studies focus upon early stages of information processing up to primary visual cortex (V1), more and more studies have started to delineate the surprisingly large number of cortical visual areas beyond V1.

Significant advances have been made in describing the functional properties of many regions in rodent cortex that process visual information. In particular, reports in mice show that several of these areas are organized hierarchically (Marshel et al., 2011; Wang et al., 2012) and functionally specialized (Andermann et al., 2011; Glickfeld et al., 2013). Anatomical and electrophysiological studies in rats have revealed many extrastriate regions that receive direct input from V1 and show retinotopical organization using electrophysiology (Montero et al., 1973; Espinoza and Thomas, 1983; Thomas and Espinoza, 1987) or anatomical methods (Olavarria and Montero, 1984; Malach, 1989; Vaudano et al., 1991; Coogan and Burkhalter, 1993; Montero, 1993). Although naming schemes vary, areas found lateral to V1 are often referred to as lateromedial (LM), laterointermedial (LI), laterolateral (LL). Studies into other functional properties of rat extrastriate regions are rare but are much needed. The value of rodent models would increase tremendously if evidence shows that neural response patterns and functional differences between areas can be linked to behavioral performance of the animals. Up to now there is only very indirect evidence for such a relationship. For example, it was shown recently that rats are able to learn complex shape discrimination tasks in which they exhibit invariance to changes in pose, illumination, and/or position (Zoccolan et al., 2009; Tafazoli et al., 2012; Vermaercke and Op de Beeck, 2012). The behavioral capacity for position invariance might very well be based upon position invariance at the neural level, which was shown recently (Vermaercke et al., 2014b).

Here we provide a more direct test of the degree to which functional differences at the neural level are related to behavioral performance. As a working hypothesis, we would expect that the functional hierarchy and specialization in rodent visual cortex reflects how the representational format of information is changed into a format which is useful for making behavioral decisions, as is assumed by current models of vision in primates (Dicarlo and Cox, 2007; Pinto et al., 2008). If this hypothesis is true, then we expect that behavioral performance would be correlated with neural selectivity in non-primary cortical areas, more than with neural selectivity in primary visual cortex.

The experiments reported here attempt to make a first step toward answering these questions. We characterized the ability of rats to discriminate pairs of shapes in a behavioral two-alternative forced choice task. Subsequently, the same stimulus set was presented to naive, awake animals while neural responses in five cortical areas (V1, LM, LI, LL, and TO) were recorded for the same set of shapes. Our results show that neural selectivity for shape differences in lower layers of extrastriate visual areas, but not in upper layers of primary visual cortex, is related to behavioral discrimination performance.

## Materials and methods

This is the primary report of the behavioral experiment, for which we provide all experimental details. The neurophysiological data were first reported elsewhere (Vermaercke et al., 2014b). The current description of these data focuses upon the most relevant aspects and new analyses in order to relate these neural recordings to the outcome of the behavioral study.

### Animals

The behavioral experiment included 12 FBN F1 rats (F1-Hybrids, first generation offspring of crossing the Fisher and Brown-Norway strains). They were obtained from Harlan animal research laboratory (Hsd, Indianapolis, Indianapolis) at an age of 5 months and were housed in groups of six per cage, further referred to as two batches of six animals. For identification, we colored each rat's tail with 1 to 6 circles using a black marker. All procedures for animal housing and testing were approved by the KU Leuven Ethical Committee for animal experiments and were in accordance with the European Commission Directive of September 22nd 2010 (2010/63/EU).

### Behavioral experiments

#### Behavioral setup

**Figure 1:**
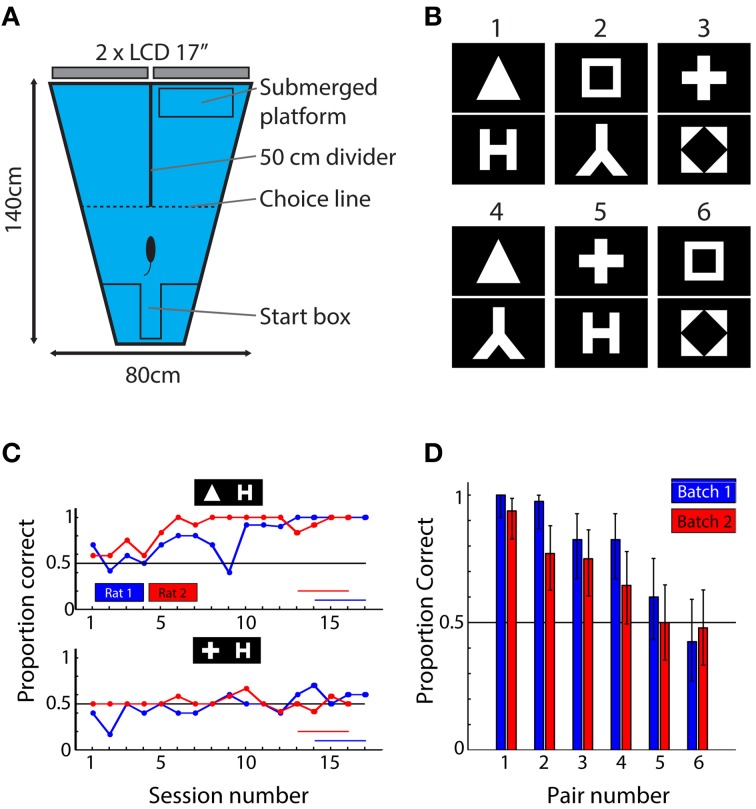
**Behavioral setup, stimuli, and results. (A)** Top-view of the behavioral setup. Animals were released at the short end of the water maze and had to find the submerged platform in one of the two arms. The identity of the shapes presented on the screens at the end of each arm predicted the platform location. **(B)** The six shape pairs used in the behavioral experiment. **(C)** Learning curves for one pair (triangle vs. letter H) that was readily learned and one pair (plus vs. letter H) that was not learned in the allocated time. Red and blue horizontal lines indicate sessions that were used to calculate mean performance. **(D)** Mean performance over the last four sessions of the experiment ordered according to average performance per pair (i.e., over two animals). Blue bars indicate performances of rats from batch 1; red bars show data for rats from batch 2. The results show that the six pairs used in this study yield a wide range of performances. Error bars indicate binomial confidence intervals at the 0.05 level.

For the behavioral task, we implemented the visual water-maze setup (V-Maze) described previously (Prusky et al., 2000; Wong and Brown, 2006; Vermaercke et al., 2014a). The setup consisted of a trapezoid pool, filled with transparent water at 26°C, and two screens (Dell 17″ LCD monitors, 1024 × 768@60 Hz) placed at the long end of the pool (Figure 1A). The animal was released into the water at the short end of the pool. From there, it has to find a submerged platform located in front of one of two screens. The reflection of the stimuli on the water obscured the platform. The rats had to learn which of two stimuli predicts the location of the platform. A 50 cm long divider was placed in between the two screens to force the animal to make a choice at that point. When crossing this point, we scored the trial as correct or incorrect depending on the location of the platform. Scoring was automated using online analysis of video images (Logitech Webcam Pro 9000) implemented in Matlab and allowing continuous tracking of the animal's position. All animals had to stay in the water until the platform was found. After a wrong decision, they were left on the platform 15 s longer. After being taken out of the water, a rat was placed under a heating lamp. Its turn for a next trial would come after all other rats of the batch completed a trial.

#### Stimuli

For studying shape processing, we selected 6 of the 8 shapes from the study by Lehky and Sereno (2007): a square, a diamond in a square, a triangle, the letter lambda, the letter H and a plus sign (Figure 1B). The exact choice of the stimuli was decided based upon the neuronal responses in inferior temporal cortex (IT) as obtained by Lehky and Sereno and included those shapes that displayed the largest variability in neural discriminability according to their data. The luminance level of each shape (i.e., the number of white pixels) was equalized. The mean width of the bounding box (the minimal rectangle containing all white pixels) around each shape was 27.3°, ranging from 23 to 33°. Stimuli were presented on a black background filling the entire display. The length of the divider determined the maximal size of the stimulus; at this point the animals had to make decision (see further). These shapes are able to drive populations of monkey anterior inferior temporal neurons, an area in monkeys which is considered as the final stage of processing in the ventral stream. At the same time they are simple black and white stimuli that contain most information in the lower spatial frequencies. This allows processing by the rat visual system with its limited visual acuity.

#### Shaping phase

This phase was not part of the actual experiment, but was meant to familiarize the animals with the setup and the goal of the task: finding the location of the submerged platform. We used two very easy stimuli (black vs. white screen), of which one (the white screen) was consistently associated with the platform. In the first trials we released the animal right in front of the platform. In this phase they had to learn that a platform can be found somewhere and that this is the only way out of the water maze. Consequently, we released them gradually further away from the screen, until they were placed beyond the divider. At this time, the animal had to make a decision in which arm to look first. The position of the white screen and associated platform (left or right) was pseudo randomized by starting at a random position in the following scheme LRLLRLRR (Prusky et al., 2000). All rats learned to solve this task after a week of two times 10–12 trials per day.

#### Experimental phase

After the animals were used to being put in the water and searched for the platform readily and consistently picked the correct side in the shaping phase, transition was made to the actual experiment. In this experiment, each animal was presented with one pair of shapes. The behavioral experiment included six of the 15 possible pair-wise combinations of the six shapes. We selected three shapes as targets and combined them with either a dissimilar or a similar distractor, with (dis)similarity derived from the dissimilarity matrix obtained for area IT by Lehky and Sereno. As a result, we obtained three hypothetically easy and difficult pairs, which would maximize the variability in behavioral discrimination performance of these pairs if discriminability in monkeys would be fully or partially related to discriminability in rats.

The experiment included two batches of six rats. We performed the experiment until the average performance across all six rats in a batch was above 70% correct for at least four successive 10 or 12-trial sessions. This criterion was chosen fairly low because we were looking for differences in difficulty between shape pairs so we expected some pairs to be more difficult and not result in a learning curve yet. With a criterion of 70%, there is ample room for individual shape pairs to be associated with much lower or much higher performance than the criterion. The two batches needed respectively, 17 and 16 sessions to reach criterion. We calculated proportion correct trials over the last 4 sessions for each pair (this proportion is further referred to as behavioral performance or BEH).

### Physical similarity measures

We obtained measures of physical (dis)similarity for these shapes based on pixel-wise or Euclidean distances (PIX) between pairs of shapes, defined as the number of pixels with a different value (binary: black or white) in the two shapes using the formula:

$$Pix_{nm}=\sum \sum \sqrt{{\left(S_{n}-S_{m}\right)}^{2}}\;m>n$$

where *n* and *m* indicate indices of different stimuli and the double sum operates over rows and columns of the resulting difference matrix. These values were then normalized, by dividing by the maximum, and rescaled to fit between 0.5 and 1 by dividing by 2 and adding 0.5. We also determined the response of a population of simulated V1 neurons (V1Sim). For this we used a simplified version of the approach described in Pinto et al. (2008). We first smoothed the images (768 by 1280 px) using a Gaussian low-pass filter (FWHM = 20px, ≅ 1.5cpd, the approximate acuity of our rats; see Prusky et al., 2002) and normalized to have zero mean and unit standard deviation. Next, the images were convoluted with 80 filters (a combination of five frequencies: 0.04, 0.08, 0.15, 0.30, and 0.60 cpd (Girman et al., 1999), and 16 orientations encompassing the full circle), with the size of each filter adjusted to include two cycles. All filters were normalized to have zero mean and norm one. The resulting response matrix *R* was compared between the 15 possible pairs of shapes and we calculated discriminability *D* as:

$$D_{nm}=1-corr\left(R_{n}\left(i,j,f\right),R_{m}\left(i,j,f\right)\right)\;m>n$$

where indices *n* and *m* refer to one of the six images, and index *f* refers to one of the 80 filter response planes. Indices *i* and *j* refer to image pixels in each filter response plane. The 15-element vector of *D*-values is rescaled to fit between 0.5 and 1 as before and will be further referred to as V1Sim.

The pixel-based distance (PIX) and the simulated V1 distance (V1Sim) were highly correlated across all shape pairs (*r* = 0.899, *p* < 0.0001; *N* = 15 shape pairs), indicating that for this stimulus set the calculation of physical dissimilarity is not very sensitive to the particular method and parameters used.

### Electrophysiological experiment

The primary report of the neural data is provided by Vermaercke et al. (2014b). Here we focus upon one experiment (“Experiment 4: Selectivity for moving shapes”) from that study which included the same stimuli as the behavioral study.

#### Animal preparation and surgery

**Figure 2:**
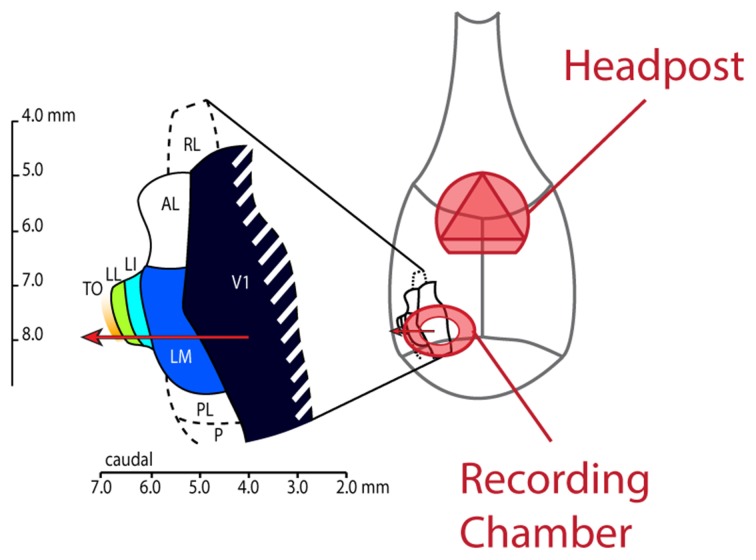
**Schematic drawing of the rat skull with locations of implanted headpost and recording chamber and layout of lateral visual areas.** This figure shows how our implants were laid out on the rat skull. The headpost was placed over bregma to leave enough room for the recording chamber and ample skull surface to attach dental acrylic. We made the craniotomy at AP −7.90 and ML 3.45 and centered the recording chamber over these coordinates. The resulting electrode track (red arrow) would typically enter cortex in the binocular part of V1 and would subsequently traverse areas LM, LI, LL and TO.

All experiments and procedures involving living animals were approved by the Ethical Committee of the university and were in accordance with the European Commission Directive of September 22nd 2010 (2010/63/EU). As also described by Vermaercke et al. (2014b), we performed microelectrode recordings in awake hybrid Fischer/Brown Norway F1 rats (*n* = 9 males), obtained from Harlan Laboratories, Inc. (Indianapolis, IN). Rats aged between 3 and 12 months, anesthetized using ketamine/xylazine, received a stereotaxically positioned 2 mm diameter circular craniotomy at −7.90 mm posterior and 3.45 mm lateral from bregma. In most animals (*N* = 6), a metal recording chamber with a base angle of 45° was placed on top of the craniotomy. A triangular head-post was fitted on top of bregma (see Figure 2). In three animals, V1 was entered orthogonally to the cortical surface, at the same location. This enabled us to record from all cortical layers in V1. A CT scan of the head confirmed the position of the recording chamber and craniotomy. Buprenorphine (50 μg/kg, i.p.) was administered postoperatively every 24 h as long as the rat showed signs of pain. When the animal was comfortable with being head restrained for at least 1 h and 30 min, we started with our recording sessions.

#### Electrophysiological recordings

As described by Vermaercke et al. (2014b), a Biela microdrive (385 μm per turn) containing a 5–10 MΩ impedance tungsten electrode (FHC) was placed on the recording chamber. For the diagonal recordings, the electrode was manually moved into the brain under an angle of 45° in steps of less than a quarter turn of the Biela drive (385 μm per full turn), thereby entering five different visual areas: V1, LM, LI, LL, and TO. Action potentials were recorded extracellularly using a Cheetah system with headstage amplifier (Neuralynx, Bozeman, MT). The signal was filtered to retain the frequencies from 300 to 4000 Hz and digitized at 32556 Hz. Action potential spikes were recorded when they crossed a threshold set well above noise level. Recordings started from the brain surface and continued until we had penetrated through the five different areas and did not find visual responses anymore or the animal started to show signs of stress. During the first few penetrations, to obtain a basic idea of the retinotopy along the electrode track, we manually determined the unit's receptive field (RF) position every 200–400 μm using continually changing shapes or small drifting circular sinusoidal gratings that could be moved across the screen. Units were recorded in all five areas at different depths, with mainly upper layers sampled in V1 and lower layers in the other areas with at least 200 μm between recording positions during a single session. Cortical depth within each area was reconstructed based on stained histological slices. For the experiment focusing on upper and lower layers in V1 using orthogonal penetrations, depth could simply be derived from the z-travel of the microdrive. Area boundaries were determined by the reflections of the retinotopic map, which were usually accompanied by obvious changes in elevation of the RF centers. A recording session generally lasted between 2 and 3 h. After removing the electrode, cleaning and capping the recording chamber, the animal was released from the head holder, and rewarded with water in its home cage (animals were water deprived prior to the recording session and only received small drops of water during recording). In each animal, we could generally perform between 10 and 15 penetrations over a period of several months. After the recording session, action potential waveforms were assigned to individual units using off-line clustering with KlustaKwik (for more details on waveform discrimination and signal quality, see Vermaercke et al. 2014b).

#### Visual stimulation during electrophysiology

Stimuli were presented to the right eye on a 24″ LCD monitor (Dell, Round Rock, Texas; 1280 × 768 pixels, frame rate = 60 Hz, mean luminance = 24 cd/m^2^, 102 × 68°) at a distance of 20.5 cm from the eye at an angle of 40° between the rostrocaudal axis and the normal of the screen. Visual stimuli were presented with custom-developed stimulation software using Matlab (The MathWorks, Natick, MA) and the Psychophysics Toolbox (Brainard, 1997; Pelli, 1997). The setup was placed within a closed, dark cabinet.

The six shapes described above were presented at identical size and contrast around the optimal position within the RF. The mean width of the bounding box around each shape was 27°, ranging from 23 to 33° (stimulus size was matched to the behavioral experiment). Because other experiments (Montero and Jian, 1995) suggested that neural responses in head-restrained animals are more sustained and more selective when stimuli are moving, the shapes were translating around this optimal RF position at four differently orientated axes of movement, separated by 45° (horizontal, vertical, and the two diagonals). The moving stimulus was shown for 4 s and the movement along each axis took 1 s. The order of the four movement axes was randomized within each 4 s presentation. During the movement along one axis, the shape started at the center (optimal) position, moved 8° (77 pixels) away from this center position in 167 ms and then moved backwards to the opposite side of the center position in 333 ms. This movement was mirrored once to complete 1 s and then the movement seamlessly continued in a different orientation. These orientations were shuffled in each trial, resulting in 24 combinations of 6 shapes × 4 orders of orientations.

### Data analysis

#### Behavior

We calculated proportion correct trials over the last four sessions for each pair and calculated 95% confidence intervals for each performance using the Matlab function *binofit* (shown as error bars in Figure 1D). We compared differences between performances for different shape pairs using permutation analysis in which we shuffled the identity of correct and incorrect trials. For these vectors, we then computed average performance correct and recorded the difference between these for each combination of pairs over 10000 iterations. When the actual difference was outside of the 95% confidence interval of this distribution, we declared the difference as significant.

#### Neural responses

For each neuron, we calculated the number of spikes elicited by each shape per trial, averaged across the 4 s stimulus presentation time. Then, we subtracted baseline activity, which was calculated as the average number of spikes in a 2 s interval preceding each stimulus presentation. Units were included when they showed a net response above 2 Hz for at least one of the shapes (i.e., non-responsive units were excluded; similar results were obtained if they were included).

To determine how well a population of neurons can discriminate between different stimuli, we implemented a linear classifier read-out similar to the one used by Rust and Dicarlo (2010) (see also Hung et al., 2005; Li et al., 2007; Vangeneugden et al., 2011). This read-out scheme is one possible way to assess the amount of information a population of units could serve to a downstream neuron, assuming this neuron applies a non-linear operation on the summed inputs. Starting with the spike count responses of a population of N neurons to P presentations of M images, each presentation of an image resulted in a response vector x with a dimensionality of N by 1, where repeated presentations (trials) of the same images can be envisioned as forming a cloud in an N-dimensional space. Linear support vector machines (SVM) were trained and tested in pair-wise classification for each possible pair of shapes (6 shapes result in 15 unique pairs). A subset of the population vectors (trials) collected for both shapes were used to train the classifier. Performance was measured as the proportion of correct classification decisions for the remaining vectors/trials not used for training (i.e., standard cross-validation). The penalty parameter C was set to 0.5 (as in Rust and Dicarlo, 2010) for every analysis.

For correlations with behavior, we retained the data for the 6 shape pairs, which were also used in the behavioral experiment.

#### Reliability and significance of SVM performance

To equalize the number of cells and trials used across visual areas, we applied a resampling procedure. On every new iteration, we selected a new subset of cells (without replacement) with the number of cells equal to the lowest number of cells recorded in a single visual area, and a random subset of trials (without replacement). We averaged over 100 resampling iterations to obtain confidence intervals for the performance. We also computed chance performance by repeating the same analysis 100 times using shuffled condition labels (thus 100 times 100 resampling iterations).

#### Chi-square and permutation analysis

We used chi-square to assess how well neural classification performance for all six pairs are matched between neural data and either physical dissimilarity or behavioral performance. We used the formula:

$$ChiSq=\sum_{i}^{n}\frac{{\left(O_{i}-E_{i}\right)}^{2}}{E_{i}}$$

where the index *i* indicates the *i*th stimulus pair of *n* pairs. *O* represents the observed values, in our case the classifier performance based on neural population responses to the *i*th pair. *E* indicates the expected values, in our study either physical dissimilarity or behavioral discriminability.

We employed permutation statistics to test the null hypothesis that the matching of shapes is not important. In order to destroy all pairwise relations, we shuffled the vector of observed values (O). We exclude shuffles that had one or more element in the original position (the pattern of results is identical without this restriction). We tested for a significant dependency between both sets of six performances by shuffling the *O*-values over all unique permutations (*N* = 265 after selection out of 720 total). *P*-values were calculated by measuring the proportion of values that are more extreme than the actually observed value.

### Permutation analysis of correlation values

We used similar procedures as described in the previous section to analyze the correlation data.

#### Data analysis to compare neural population discriminability with behavioral difficulty

Based upon earlier work in humans and other primates (Dicarlo and Cox, 2007; Op de Beeck et al., 2008), we expect a high correlation between V1 discriminability and physical, pixel-based (or V1-simulated) distances between stimuli but not between V1 and behavioral discriminability. At the same time, we expect a high correspondence between TO and behavioral discriminability, but not between TO and physical distances. To test this prediction, we constructed the transformation index H that captures this relation:

$$\begin{array}{l} H=\left[Z\left(TO,Behavior\right)-Z\left(TO,Pix\right)\right]\\ \;-\left[Z\left(V1,Behavior\right)-Z\left(V1,Pix\right)\right] \end{array}$$

where *TO* and *V1* refer to the neural population discriminability of the 6 shape pairs in area *TO* and *V1*, respectively. Behavior corresponds to animal performance on these pairs; and Pix refers to the pixel-wise difference between the shapes of these pairs. The operator Z corresponds to the sample Pearson correlation between both performances after Fisher-Z transformation. High values of the index would provide support for our assumption that there is a transition from pixel-related discriminability in V1 to behavior-related discriminability in TO. This index was also calculated for permuted data (same procedure as above) and we used the 95th percentile value as the threshold for significance of the obtained index.

## Results

### Behavioral shape discrimination

One specific hallmark of the ventral visual pathway in primates is that neural responses and neural discriminability are more related to behavioral performance for higher-level regions than for e.g., V1, where we expect more correspondence with measures of local pixel-level differences.

To test whether this is true in rodents as well, we first obtained behavioral data from 12 rats about the relative discriminability of different shape pairs. These rats were trained in a visual water task (see Figure 1A) to discriminate between different shape pairs, one shape pair per rat. Six shape pairs of the 15 possible pairs were included and two rats were trained per shape pair (see Figure 1B), one in each batch of six rats. We equated the length of training across rats/pairs. Based upon primate literature, we would expect that those shape pairs that would be associated with the best behavioral discrimination performance at the end of training would also be associated with a higher neural discriminability in the higher areas in the identified rat visual pathway, but not in area V1. Given animals have to find the target shape while moving around in a water maze, it would also be unlikely that a simple V1 representation, lacking position invariance, would be sufficient to drive the animals' decision process.

After 17 and 16 behavioral training sessions for the first and second batch respectively, average performance across all rats reached the criterion of 70% correct. We noticed clear differences in performance between the shape pairs (Figure 1C). A few shape pairs were associated with performance close to 100% correct, while two other shape pairs were associated with performance close to the chance level of 50%. This variation in performance across shape pairs generalized from the first to the second batch of six animals (blue and red bars in Figure 1D): the variation in performance across shape pairs was highly correlated between the two batches (*r* = 0.92, *P* = 0.009, *N* = 6 shape pairs). To assess whether the ordering was important, we pooled the data for two animals per pair and performed a permutation test to compare the average performance between all possible combinations of shape pairs (see Methods). We found the all differences to be significant, except for those between pair 2–3, 3–4, and 5–6. We conclude that the order matters for most pairs and that correlations based on these data are meaningful.

With the data of just the first batch of rats, it would have been conceivable that the differences between shape pairs would be related to interindividual differences between the rats, given that each shape pair was tested in a different animal. However, the near-perfect replication of the across-pair variation in performance in the second batch of animals argues against this alternative hypothesis in terms of interindividual differences. This alternative hypothesis is also not consistent with the fact that all rats had shown a very similar performance in the preceding shaping phase (mean = 0.91, *SD* = 0.06, *N* = 12), in which rats were trained in the general task layout using full field white and black stimuli. To quantify this, we paired rats that would receive the same shape pair in the next phase, calculated their average performance obtained during the last four shaping sessions and performed a paired *t*-test: [*t*_(5)_ = 0.4008; *P* = 0.7051, *N* = 6]. The small differences in performance were also not correlated (*r* = 0.05, *P* = 0.9245, *N* = 6), excluding any preexisting similarities between the rats that would explain striking similarity in performance for the shape pairs. This suggests that the two batches start out as fairly homogeneous groups that react in a consistent way when confronted with different stimulus pairs.

We quantified the average time animals needed to make a decision; this includes swim time from the start of the trial until the animal passed the divider. Median reaction times were 5.73 and 5.47 s for both batches [*N* = 1045 and 1145 trials; Q25–75 = (4.94 9.30) and (4.80 6.76) s]. These values are comparable to the presentation duration used for the electrophysiological recordings.

### Neural discrimination performance

The data described here form a subset of a larger dataset reported earlier (Vermaercke et al., 2014b). This earlier study characterized responses of single neurons and populations in rat primary visual cortex (V1) and 4 extrastriate areas (LM, LI, LL, and newly found area TO). We focus here on the extent to which each of the five defined cortical areas allows the discrimination of the six shape pairs. While showing these shape stimuli, we recorded from a total of 631 (114, 104, 166, 107, 140 for areas V1, LM, LI, LL, and TO, respectively) neurons. After selecting responsive neurons to which each shape had been presented at least 12 times, we retained 413 single units (88, 63, 131, 68, and 63; this yields 63 neurons per SVM subsampling). The percentage of responsive neurons was 77, 61, 79, 64, and 45 for areas V1, LM, LI, LL, and TO, respectively. Between neurons there was a large variation in exact receptive field position (Vermaercke et al., 2014b).

**Figure 3:**
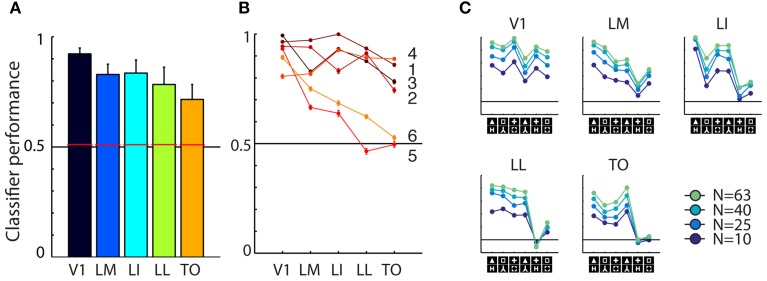
**Overview of neural data. (A)** Average SVM classification performance for the six shape pairs used in this study, for each of the five areas. Red bars indicate significance threshold based on shuffled condition labels. Error bars indicated SEM over six pairs. **(B)** Classifier performance for individual pairs, pair numbers correspond to those used in Figure 1B. Performance for pairs five and six falls to chance toward higher areas. **(C)** Results for the control analysis in which we reduced the number of units included in individual sub-samplings of the SVM classifier. Average performance decreases with lower number of cells included, but the overall pattern of classification is preserved. The order of shape pairs corresponds to that in Figures 1B,D.

Averaged across the six shape pairs, we found reasonable and strongly significant population decoding performance in every area [Figure 3A; *V1* = 92.33%, *t*_(5)_ = 15.7517, *P* = 0.0000; *LM* = 82.54%, *t*_(5)_ = 15.7517, *P* = 0.0000; *LI* = 83.53%, *t*_(5)_ = 15.7517, *P* = 0.0000; *LL* = 78.00%, *t*_(5)_ = 15.7517, *P* = 0.0000; *TO* = 71.99%, *t*_(5)_ = 15.7517, *P* = 0.0000; error bars show SEMs]. When performing the permutation analysis using shuffled trial labels, we obtain chance level estimates of which the 95th percentile is shown as red horizontal bars in Figure 3A; all corresponding *p*-values for each area fall below the 0.0001 level.

We also tested whether differences between areas reached significance by doing a similar permutation analysis using shuffled area labels. We did this for all pair-wise comparisons and found classification performances in all areas to be significantly different, except for the difference between LM and LI. All these analyses gave similar results when performed after matching the average firing rates between areas (data not shown, see Vermaercke et al., 2014b for details on a similar matching procedure). On a more detailed level of analysis, we find that performance for all shape pairs is fairly high in V1, but shows a differential pattern in higher areas (see Figure 3B). Discrimination performance for four out of six shape pairs decreases slightly over areas, for two other pairs performance decrease is stronger.

Because we are interested in correlating neural responses to behavioral performance, we performed a control analysis to rule out that correlations with area V1 would be distorted/diminished by a ceiling effect with a generally high neural discriminability. To control for this, we included a progressively lower number of cells (*N* = 63, 40, 25, 10) for each SVM resampling. This will bring down the average performance level, which would allow for a pattern that could be compressed by overall high performance, to reappear. The curves in Figure 3C confirmed that this manipulation was effective: SVM models based on a smaller amount of cells show a lower overall performance. When examining the shape of the curve, we find only minor changes in the relative differences between shape pairs.

### Correlations between pixel-based differences, neural responses, and behavior

We combined data from the behavior and electrophysiology experiment to determine which of the cortical areas are more likely to underlie shape discrimination. We also included a measure of pixel-wise differences, which captures low-level similarity of the shapes [see Methods; responses of a simulated population of V1 neurons (V1Sim) yielded highly similar results]. The correlation between PIX and behavioral performance was non-significant (*r* = 0.110, *P* = 0.84, *N* = 6 shape pairs), which potentially allows us to find differential correspondences between the neural responses and either physical properties or the behavioral output of the animal.

**Figure 4:**
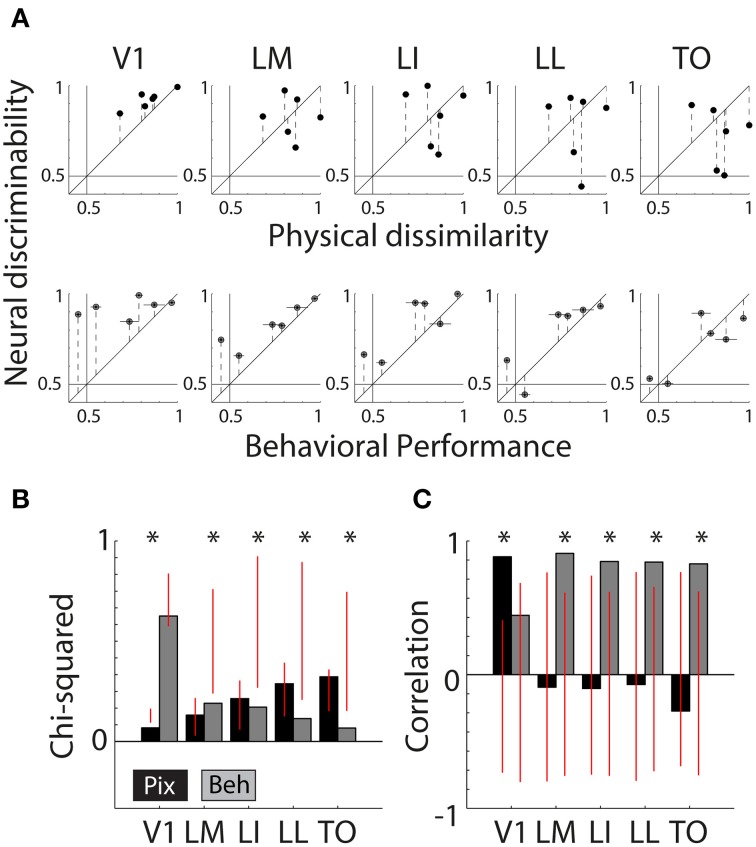
**Correlations between neural responses, pixel similarity, and behavioral performance. (A)** Scatterplots of neural discriminability data for six shape pairs in five cortical areas compared to physical dissimilarity (average between PIX and V1Sim measures; top row) and behavioral performance (average for two rats; bottom row). Correspondence with physical dissimilarity is high for V1 and decreases toward higher areas; i.e., points fall further from the diagonal identity line. In contrast, behavioral performance is increasingly well matched to neural discriminability toward higher areas, with points falling closer to the diagonal. Error bars indicate SEM over both physical dissimilarity measures in top row, SEM over both animals in bottom row and SEM for neural data in both. **(B)** Chi-square values are reported for each scatter plot shown in **(A)**. Black bars show chi-square values between physical dissimilarity and neural data. Gray bars show chi-square values for neural data and behavior. Red vertical lines indicate the random distribution obtained through permutation analysis. Stars indicate significant chi-squares values at the 0.05 level. **(C)** Correlations as a measure of correspondence of neural discriminability with pixel-based differences (black bars) and with behavioral performance (gray bars). Red vertical lines indicate the random distribution obtained through permutation analysis. Stars indicate significant correlations at the 0.05 level.

Figure 4A shows scatter plots of the neural discriminability against either the pixel-based differences (top row) or the behavioral discrimination performance (bottom row). For physical dissimilarity, we pooled both measures, PIX and V1Sim, because they were highly correlated. For the behavioral results, we pooled the performances of both animals that had to learn the same pair (error bars are calculated over both animals; individual error bars are shown in Figure 1D). On a qualitative level, the dots seem to be close to the identity line in V1 for physical measure and diverge in higher areas. The opposite trend is seen for BEH where correspondence improves drastically toward higher areas.

To quantify these effects, we use two separate measures of correspondence: chi-square (see Methods) and Pearson correlations. Both are presented with permutation statistics (see Methods).

Figure 4B shows chi-square values for both PIX (black bars, *V1* = 0.08 *p* < 0.0001, *LM* = 0.16 *P* = 0.4300, *LI* = 0.26 *P* = 0.3429, *LL* = 0.35 *P* = 0.3755, *TO* = 0.39 *P* = 0.1858) and BEH (gray bars, *V1* = 0.75 *P* = 0.1518, *LM* = 0.23 *P* = 0.0076, *LI* = 0.21 *P* = 0.0220, *LL* = 0.14 *P* = 0.0336, *TO* = 0.08 *P* = 0.0308), the red vertical lines indicate the distribution of values obtained through the permutation analysis (see Methods). If a bar is outside of the overlaid red line, the observed value is significant and we can reject the null hypothesis that the order of pairs is not important. This shows us that neural responses patterns in V1 and PIX are more similar than expected by chance. The neural responses in the four extrastriate areas show a significant correspondence with BEH.

In Figure 4C we show the correlation values for both PIX (black bars, V1 *r* = 0.88, *P* = 0.02; LM *r* = −0.09,*P* = 0.86; LI *r* = −0.10,*P* = 0.85; LL *r* = −0.07,*P* = 0.89; TO *r* = −0.27,*P* = 0.60) and BEH (gray bars, V1 *r* = 0.44,*P* = 0.38; LM *r* = 0.91,*P* = 0.01; LI *r* = 0.85,*P* = 0.03; LL *r* = 0.84,*P* = 0.04; TO *r* = 0.83,*P* = 0.04), again the red vertical lines indicate the distribution of values obtained through the permutation analysis (see Methods). The correlation with PIX is only significant for neural data in V1, while BEH correlates significantly with response pattern obtained in extrastriate areas. Here we report correlations including only the six shape pairs used in the behavioral experiment. The correlations with pixel-based differences show a very similar pattern when calculated using data from all 15 possible shape pairs (these correlations are reported in Vermaercke et al. (2014b).

Thus, Chi-square values and correlations show a consistent effect for TO compared to V1, with a strong correlation between neural discriminability and behavioral performance in TO and no correlation in V1. For Chi-square values the change from V1 and TO seems to occur gradually, with intermediate results in the intermediate brain regions, while for correlations all non-V1 areas have a strong correlation with behavior.

We did not make a priori predictions about the nature of shape representations in intermediate areas along the pathway. Predictions were very clear-cut, however, for how the representation of shape should be different when comparing the two extreme areas: we expected V1 neural discriminability to correlate well with pixel-based stimulus differences, and TO neural discriminability to correlate well with behavioral performance. We constructed a “transformation index” that captures this shift in the nature of shape representations in one value (see Methods). This index essentially results in one number that tells us how the similarity of neural responses to stimuli (PIX) and behavior (BEH) changes from V1 to TO. For chi-square, this value is -0.9763, outside of the range (−0.0399 0.0434) and significance *p* < 0.001. For correlations, the significance of the empirically observed transformation index [2.37, outside of the range (−2.1930 1.7294)] was *p* < 0.05. Thus, the prediction of a transformation in how shape is represented from V1 to TO was confirmed by the data.

### Fine transition of representations in V1

We performed a similar analysis with the V1 data from the orthogonal penetrations in which we distinguished between upper and lower layers. This is a relevant additional dataset because in the diagonal recordings the V1 data are biased toward the upper layers (see Vermaercke et al., 2014b). Thus, we can consider upper-layer recordings in the orthogonal penetrations as a replication attempt of the results from the diagonal penetrations, while the lower-layer recordings provide new data to test whether there is already a transformation of shape selectivity within V1.

**Figure 5:**
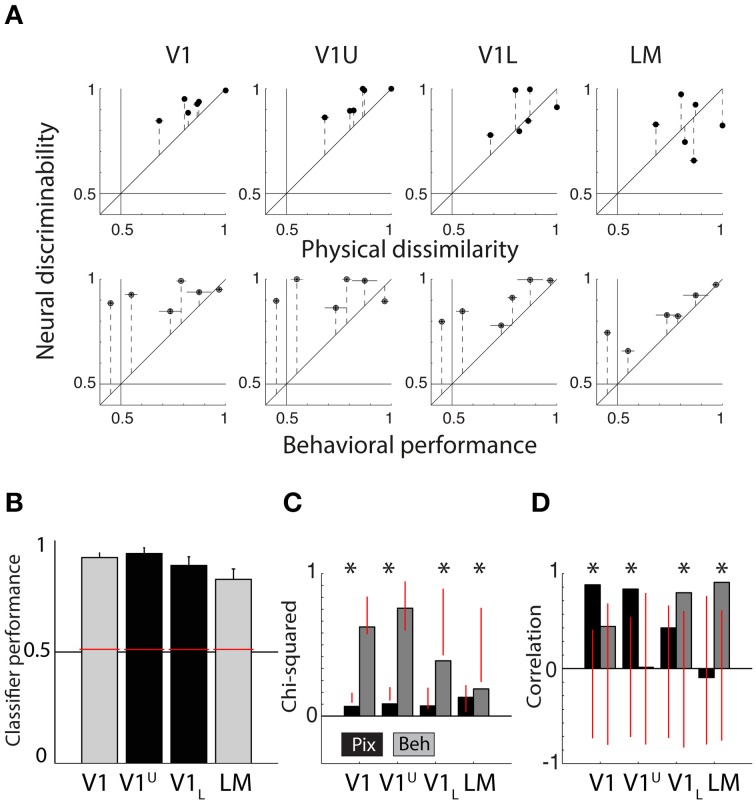
**Summary of neural data collected in upper and lower layers of V1. (A)** Scatter plots showing neural discriminability of six shape pairs in upper and lower layers of V1 relative to physical dissimilarity (average between PIX and V1Sim measures; top row) and behavioral performance (average for two rats; bottom row). Results from Figure 4 of the V1 and LM recordings from the diagonal penetrations are re-plotted here for visual comparison. Error bars indicate SEM over both dissimilarity measures in top row, SEM over both animals in bottom row and SEM for neural data in both. **(B)** Average SVM classification performance for the six pairs in upper layers of V1 (V1U, shown in black) and lower layers of V1 (V1L, shown in black). We also show the data for V1 and LM (gray bars) shown in the Figure 3A, for comparison. Overall performance is high in all four areas. Error bars show SEM for six shape pairs. **(C)** Chi-square values for the scatterplots shown in **(A)**. V1U results are very similar to the results obtained in V1 in the diagonal recordings. V1L results fall in between V1 and LM from the diagonal recordings. Red vertical lines indicate the random distribution obtained through permutation analysis. Stars indicate significant chi-squares values at the 0.05 level. **(D)** The correlations for the data shown in **(A)**. Again, V1U is more comparable to the data we collected in V1 during diagonal recordings and V1L forms an intermediate step in between V1 and LM. Red vertical lines indicate the random distribution obtained through permutation analysis. Stars indicate significant correlations at the 0.05 level.

We classified all units beyond a depth of 500 micron as belonging to the lower layers (for more information, see Vermaercke et al., 2014b). We recorded in total from 131 neurons in three animals (V1 Upper or V1U: 61, V1 Lower or V1L: 70). After selection based on responsiveness and number of trials, we retained 44 units in V1U and 57 units in V1L. Using these new (non-overlapping) V1 data we find a further finer transition within V1. In terms of average classifier performance for the six shape pairs, both subdivisions of V1 achieve high scores (V1U = 94.11%, V1L = 88.78%, see Figure 5B; the data for V1 and LM from the previous section are replotted for reference, colors match those in Figure 3A). When we run our permutation analysis on the scatterplot data shown in Figure 5A, we find that V1 and V1U show significant chi-square values for PIX (V1: *P* = 0.0138, V1L: *P* = 0.0055, see Figure 5C), while V1L and LM show significant chi-square values for BEH (V1L: *P* = 0.0507, LM: *P* = 0.0151).

The correlations between neural responses in V1 and V1U and PIX show a similar pattern (V1: *r* = 0.8821, *P* = 0.0140, V1U: *r* = 0.8367, *P* = 0.0062, see Figure 5D), and V1L and LM relate more to BEH (V1L: *r* = 0.7982, *P* = 0.0202, LM: *r* = 0.9072, *P* = 0.0106). Parametric tests show that the correlation between V1L and BEH is not significant (*P* = 0.057), which contradicts the result from the permutation statistic, so it would be prudent to say that the lower layers in V1 may form an intermediate step between upper layers in V1 and LM.

The pattern that emerges is that the representation in the upper layers is most similar to PIX/V1Sim while lower layers are already shifted partially toward the extrastriate regions (see Figure 5A). One interpretation could be that upper layers receive information from thalamus and after initial processing, transmit it further to downstream areas. After this first step of information reformatting, neural discriminability in V1L starts to resemble behavioral performance and this becomes even clearer in area LM.

## Discussion

We obtained a behavioral measure of shape similarity from two independent groups of rats. We also recorded neural responses to individual stimuli in yet another group of naïve rats. Taken together, both datasets allowed us to determine which cortical area is most likely to underlie behavior. As expected, primary visual cortex encodes the stimuli in terms of simple features, which is well captured by pixel similarity and convolution-type models. Higher areas show more similarity to behavioral responses, with highest area TO showing the best fit. These results indicate that visual information is transformed from representing simple features to a representation that is used to drive behavior, a process reminiscent of ventral stream in non-human primates (Op de Beeck et al., 2001; Dicarlo et al., 2012). As reported by Vermaercke et al. (2014b), neural responses in area TO also tend to be most robust to changes in stimulus position, which would make these responses more reliable to be used in behavioral decision making. At least to some degree, invariance is needed to complete a swim trial, so performance would not be expected to depend purely on physical differences between stimuli. The representation of a shape in V1 is highly dependent on it's retinal position, which changes drastically during swimming. Basing performance on the population response in V1 would be sub-optimal during a swim task, even though it has a better capability of reliably encode patterns in the outside world. At least in our untrained animals, the neural data show that even though responses to shapes are reduced in higher areas, the representation becomes more informative to the task as populations of neurons in these areas prefer the same shape in different positions.

The previous reported work of Vermaercke et al. (2014b) examines many properties of neuron in multiple areas along a diagonal track through lateral visual cortex. Based on retinotopy, latency and to some extent, receptive field size, they defined five different areas. Using neural responses elicited by the six shapes, they were able to characterize that the representation of information changes over areas. Moreover, by presenting stimuli at different positions within the receptive field, they found evidence for increasing generalization performance for the same shape at the other position, indicative of position tolerance. Taken together, these data indicate that the five areas are part of a hierarchical network that may be involved in shape processing. The current study focuses on a subset of the shape pairs to investigate how well naïve animals would be able to differentiate between them at the behavioral level. By quantifying the representations in each of the areas, we were able to pinpoint some of the transformations the visual information undergoes. There appears to be a sharp transition between areas V1 and LM, however, as shown in Table 1 of Vermaercke et al. (2014b), the pattern of transition between areas depends on what feature is being investigated. Some features show a stepwise pattern (not always V1 vs. other areas), other properties (e.g., orientation tuning) change gradually over areas.

The present study is obviously limited by the simplicity of the stimuli used, and the low number of different stimuli. Future studies should be conducted with more stimuli and with more stimulus pairs. This would require a more automated setup, unlike the labor-intensive visual water maze used in the present study. Typically, rats show relatively fast learning curves and high accuracy rates in this visual discrimination water maze, more so than often obtained in tasks using liquid or food rewards (Zoccolan et al., 2009; Meier et al., 2011; Tafazoli et al., 2012; Vermaercke and Op de Beeck, 2012; Alemi-Neissi et al., 2013). The level of motivation might be considerably higher when animals have to escape from a water tank. Despite these benefits, the visual-water task includes a low number of trials per session, and each trial has to be started manually by the experimenter. This limits the number of stimuli for which reliable performance estimates can be obtained.

Future studies could make use of parametric stimulus sets that are constructed to test specific predictions on how rats process visual objects (e.g., rotated views of objects, morphs between two know prototypes, different classes of objects etc.). Here we correlated behavioral data with neurophysiological recordings in other, naïve animals. Ideally, future studies would perform the neural recordings during the execution of the behavioral task so that direct and more causal relations between neural responses and behavioral outcomes can be investigated.

As a follow-up to the present study, we continued training with the first batch so that all animals eventually were trained in all six pairs followed by a recall phase in which performance for all pairs was checked. The data from this further testing are hard to interpret because of interference between the different shape pairs (e.g., already higher than chance performance on the first day of a new pair), but in the present context it is relevant that average performance in this recall phase was well above 70% correct, for each animal (75.86, 78.82, 83.30, 86.08, 79.68%). For one pair (+ vs. H) performance was still rather low (66.34%), suggesting that it might be extremely hard for the rats to disentangle the representations of both stimuli. Nevertheless, discrimination performance was above chance even for this pair, indicating that all the shape pairs can eventually be learned by the animals, most likely even up to close to 100% correct with long enough training. For further work it would be interesting to investigate the neural representation in these areas during and after training. Using chronically implanted electrodes or two-photon imaging, it should even be possible to monitor neural population and to characterize how the representations in the different cortical areas are changing due to the training. Causal manipulations within the same animal (e.g. lesions, optogenetics or pharmacology) will be crucial in shedding light on the importance of each visual area for shape processing and behavior.

### Conflict of interest statement

The authors declare that the research was conducted in the absence of any commercial or financial relationships that could be construed as a potential conflict of interest.
